# Degradation of Low-Molecular-Weight Diesel Fractions (C_10_−C_16_ Alkane) Drives Cd Stabilization and Pb Activation in Calcareous Soils from Karst Areas

**DOI:** 10.3390/toxics13060496

**Published:** 2025-06-13

**Authors:** Yiting Huang, Yankui Tang, Zhenze Xie, Jipeng Wu, Jiajie Huang, Shaojiang Nie

**Affiliations:** 1 School of Civil Engineering and Architecture, Guangxi University, Nanning 530004, China; huangyitingpp@163.com; 2 School of Resources, Environment, and Materials, Guangxi University, Nanning 530004, China

**Keywords:** co-contaminant, chemical forms, Pearson correlation analysis, DOM, aging process, sterile soils

## Abstract

The influence of petroleum hydrocarbons (PHCs) on the transport and transformation of heavy metals may limit bioremediation efficiency. The mechanisms by which PHC degradation intermediates control heavy metal distribution in calcareous soils from karst areas require further exploration. This study systematically investigated how compositional changes in diesel fuel during aging regulated the fate of Cd and Pb in calcareous soils. The results demonstrated that the low-molecular-weight fractions of diesel fuel (C_10_−C_16_) were preferentially degraded. This degradation process altered zeta potential, cation exchange capacity (CEC), and pH, thereby promoting Cd stabilization through electrostatic attraction and speciation transformation. Particularly, reducible Cd content showed a strong positive correlation with C_16_ content (*r* = 0.88, *p* < 0.05). Furthermore, the degradation of C_10_−C_16_ fractions caused Pb transformation from residual to bioavailable fractions by stimulating microbial activity. Residual Pb content was positively correlated with C_10_−C_16_ fractions (*r* = 0.55, *p* < 0.05). Notably, dissolved organic matter (DOM) and CaCO_3_ content in calcareous soils enhanced Cd and Pb adsorption, thereby weakening the interactions between these metals and C_10_−C_16_ fractions. Consequently, multiple linear regression (MLR) models relying exclusively on C_10_−C_16_ degradation parameters showed poor fitting coefficients for Cd/Pb mobility. The present work provides scientific guidance for heavy metal bioremediation in calcareous soils.

## 1. Introduction

Heavy metal contamination, originating from both natural accumulation and anthropogenic emissions, has emerged as a critical global environmental issue. These metals, particularly cadmium (Cd) and lead (Pb), pose severe threats to human health through the food chain and dermal contact [1]. The demand for reusing land is driven globally by increased urbanization, leading many policymakers to prioritize the remediation of heavy-metal-contaminated soils. Specific microorganisms can be employed for mitigating heavy metal toxicity through biosorption, biotransformation, and biomineralization processes [2,3]. Such microbial-mediated remediation is widely recognized as a sustainable and cost-effective approach. However, heavy metals tend to migrate away from their original sources due to gravitational forces and rainfall infiltration. This dispersion reduces the targeted delivery efficiency of nutrients and exogenous microorganisms in bioaugmentation or biostimulation. On the other hand, the toxicity of heavy metals significantly influences the growth and metabolism of microbial communities [4,5]. To develop effective bioremediation strategies, a comprehensive understanding of the transport and transformation of heavy metals in soil is essential.

Previous studies have shown that the fate of heavy metals in soils is significantly influenced by factors such as soil pH, ionic competition, and the physicochemical properties of the heavy metals [6,7]. Notably, heavy metals commonly co-occur with petroleum hydrocarbons (PHCs) in soils as a result of wastewater irrigation, industrial processes, and atmospheric deposition [8,9,10,11]. Previous studies have demonstrated that the presence of PHCs can decrease the bioavailability and toxicity of heavy metals by (1) altering microbial membrane permeability, (2) interfering with molecular binding functions, and (3) modulating enzyme catalytic activity [5,12]. Microbial communities consume PHCs and subsequently decrease soil pH through CO_2_ production [13,14]. During the natural attenuation, PHCs can alter dissolved oxygen (DO), SO_4_^2−^, and HCO_3_^−^ in soil environments [14,15]. These changes may facilitate the transformation of heavy metals into stable or bioavailable forms [16,17,18]. Furthermore, PHCs shield charges on soil surfaces, directly altering the soil’s zeta potential [19]. Particularly, high concentrations of PHCs may form cation–π bonds with Pb [20]. These effects ultimately modify the transport behavior of heavy metals in soil [7,21]. While previous studies have documented the general effects of petroleum hydrocarbons (PHCs) on heavy metal fate, systematic investigation of how degradation-induced PHC compositional changes affect this process is still lacking.

Due to the presences of PHCs, conventional remediation technologies (such as bioremediation) often struggle to achieve satisfactory removal of heavy metals, resulting in the long-term persistence of both pollutants in soils [22]. Subsequently, PHCs degrade into intermediates, which have altered molecular structures and exhibit different toxicity, bioavailability, and mobility compared to the parent compounds [5,23,24,25]. These intermediates typically coexist with heavy metals, but their role in controlling the transport and transformation of heavy metals remains unclear. Moreover, calcareous soils in karst regions are characterized by high pH, high soil organic matter (SOM) content, and abundant Ca^2^⁺ [26]. These properties may promote the degradation of PHCs in soils [27]. Unfortunately, the effects of PHC degradation characteristics on heavy metal speciation in these soils remain poorly understood.

Diesel fuel, with alkanes as its major constituents (70–85% [28]), represents a global environmental challenge when spilled. In this study, diesel-fuel-contaminated soil was selected as a representative scenario of PHC pollution, with a focus on its C_10_−C_31_ alkanes (abbreviated as C_10_−C_31_). The aims were to (1) characterize how changes in the composition of C_10_−C_31_ in diesel fuel during the aging process affect the transport and transformation of Cd and Pb in calcareous soils; (2) evaluate the impact of soil properties in calcareous soils; and (3) establish statistical relationships between Cd, Pb, and C_10_−C_31_ concentrations. The results provide a clear understanding of heavy metal fate in PHC-contaminated calcareous soils, guiding a scientific basis for developing bioremediation strategies in karst environments.

## 2. Materials and Methods

### 2.1. Reagents

Diesel fuel (0#) (density: 0.84 g/mL) was obtained from a commercial market in Nanning, China. Its composition by weight was 82.9% alkanes, 17.09% aromatic hydrocarbons, and <0.035% sulfur. Cadmium chloride (CdCl_2_) and acetone were obtained from Kermel-Chemical Co., Ltd. (Shanghai, China) and Damao-Chemical Co., Ltd. (Tianjin, China), respectively. N-hexadecane (C_16_H_34_. Abbreviated as C_16_) was purchased from Macklin-Biochemical Co., Ltd., Shanghai, China.

All regents were at least analytical grade.

### 2.2. Soil Incubation

Two types of calcareous soils in karst areas were selected in this study: black calcareous soils (BKC) and brown calcareous soils (BWC). These soils were sourced from a depth of 0–20 cm below the ground surface in Fusui County, Guangxi Zhuang Autonomous Region, China (22°56’43.6″ N, 107°15’46.7″ E). This sampling site is characterized by an extensive karst landscape [29]. Importantly, BKC and BWC were confirmed to be free from pesticides, nitrate pollution, and PHCs. These soils were first dried at 25 ± 1 °C for one week in the laboratory, then sieved through a 2 mm sieve. The properties of BKC and BWC are presented in Table S1.

Diesel fuel was selected as a representative PHC in soils. This study specifically focused on its dominant alkane components (C_10_−C_31_) [28]. During long-term natural attenuation of diesel fuel, n-hexadecane (C_16_H_34_, abbreviated as C_16_) persists in soil, even post-remediation [30]. Therefore, C_16_ was employed as a model intermediate degradation product to further examine the impact of residual diesel components on heavy metal transformation. The physicochemical characteristics of diesel fuel and C_16_ are presented in Table S2. Furthermore, as Cd is recognized as the most concerning heavy metal in PHC-contaminated sites, it was chosen as the model heavy metal in this study [31].

To simulate co-polluted soils, both BKC and BWC were artificially spiked with 20 mg/kg Cd and 4500 mg/kg diesel fuel (or C_16_), following the limits specified in the Soil Environmental Quality Risk Control Standard for Soil Contamination of Development Land in China [32]. Based on methods documented in prior research [33,34], the soil incubation was conducted as follows: (1) Diesel fuel (or C_16_) and CdCl_2_ were dissolved in a mixture of acetone and Milli-Q water (1%, *w*/*w*) to prepare a stock solution. The solution was then stirred continuously for 24 h using a magnetic stirrer to ensure homogeneous mixing. (2) Next, 150 mL of the stock solution was added to 1.0 kg of BKC with continuous stirring, resulting in final concentrations of 20 mg/kg of Cd and 4500 mg/kg of diesel fuel (or C_16_) in the soil. The same procedure was repeated for BWC to ensure identical contamination levels. (3) All contaminated soils were aged in the dark at 25 °C, with soil moisture content maintained at 15 % (*w*/*w*) through weekly replenishment with Milli-Q water [35]. The contaminated soils were aged for 30 and 90 days to capture alterations in diesel fractions. In this work, soils contaminated for different durations were packed into separate columns. (4) To reduce the influence of moisture content on the results, uncontaminated calcareous soils were also maintained at 15 % (*w*/*w*) soil moisture.

The properties of soil samples were determined using the methodologies provided in Text S1.

### 2.3. Column Setup

The study employed a polyethylene glycol terephthalate (PET) column with an inner diameter of 6.0 cm and a length of 12.0 cm. These dimensions were specifically chosen to emphasize vertical migration of contaminants while suppressing lateral diffusion effects. Furthermore, five 0.2 cm diameter drainage holes were uniformly installed at the column base.

**Figure 1:**
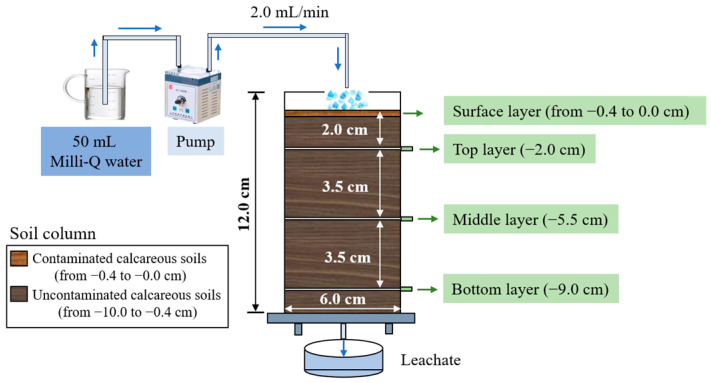
Schematic of the experimental setup.

As shown in Figure 1, the column was packed in the following sequence: (1) A 74.0-μm plastic mesh served as a soil particle retention barrier and was placed at the column base. (2) Next, 285.0 ± 0.1 g of uncontaminated soil was filled from 10.0 cm to 0.4 cm below the ground surface (0.0 cm). (3) After that, 30.0 ± 0.1 g of artificially contaminated soil (Section 2.2) was evenly distributed in a layer spanning −0.4 cm to 0.0 cm. (4) An additional 74.0 μm plastic mesh was placed on the 0.0 cm surface to prevent soil splash. (5) Finally, the packed column had a bulk density of 1.12 g/cm^3^.

Experimental groups are presented in Table 1. Each experimental group comprised three parallel columns, with strict control of soil weight and layer thickness (Table S3).

### 2.4. Leaching Experiments and Sample Analysis

The leaching parameters (including intensity, frequency, and duration) were set based on the characteristics of extreme precipitation events in karst regions [36,37,38]. Specifically, 50 mL of Milli-Q water was injected into the packed column from the top to the bottom using a pump at a flow velocity of 2 mL/min (Figure 1). This leaching process was performed once a week and was conducted a total of three times.

After each leaching process, soil samples were collected from the top (2.0 cm), middle (5.5 cm), and bottom (9.0 cm) layers below the ground surface (0.0 cm) (Figure 1). Leachate was collected only after the 3rd leaching process. Subsequently, the fractions and concentrations of C_10_−C_31_ and Cd in soil and leachate samples were analyzed following the procedures detailed in the Supplementary Materials (Texts S2 and S3). Although Pb was not added to the soils, its concentrations and chemical forms were quantified given its natural prevalence in calcareous soils and as a co-contaminant in PHC-impacted soils [8,39]. The Pb measurement methods are described in Text S3.

### 2.5. Statistical Analysis

Analysis of variance (ANOVA), coupled with Duncan’s test (at the *p* < 0.05 level), was performed to investigate significant differences in the levels of C_10_−C_31_, Cd, and Pb among the experimental groups. Principal component analysis (PCA) was used to identify key fractions of C_10_−C_31_ associated with the chemical forms of Cd and Pb in calcareous soils, followed by Pearson correlation analysis to examine the relationships between these variables. Furthermore, assuming linear relationships between variables can provide a baseline for initial trend estimation. This assumption is further supported by the potential linear adsorption–desorption behavior of contaminants under equilibrium conditions, especially at low concentrations. Subsequently, multiple linear regression (MLR) was applied to quantify Cd and Pb transport during C_10_−C_31_ aging, with performance verified by results.

ANOVA and MLR were performed using SPSS 22.0 software, while PCA and Pearson correlation analysis were conducted with Origin 2021. Graphical representations of mean values were created in Origin 2021.

## 3. Results and Discussion

### 3.1. The Distribution of Cd and Pb in Diesel-Fuel-Contaminated Soils

Based on previous studies [24,40], the C_10_−C_31_ was categorized into (a) C_10_−C_16_ as the low-molecular-weight fraction, (b) C_17_−C_21_ as the medium-molecular-weight fraction, and (c) C_22_−C_31_ as the high-molecular-weight fraction. Their distribution was then analyzed. As the aging period progressed from 30 to 90 days, C_22_−C_31_ maintained higher residual concentrations in the surface contaminated layer (−0.4 cm to 0.0 cm) of Col. 1–Col. 4 (Table S4), due to their lower bioavailability, volatility, and hydrophilicity [25,41].

**Figure 2:**
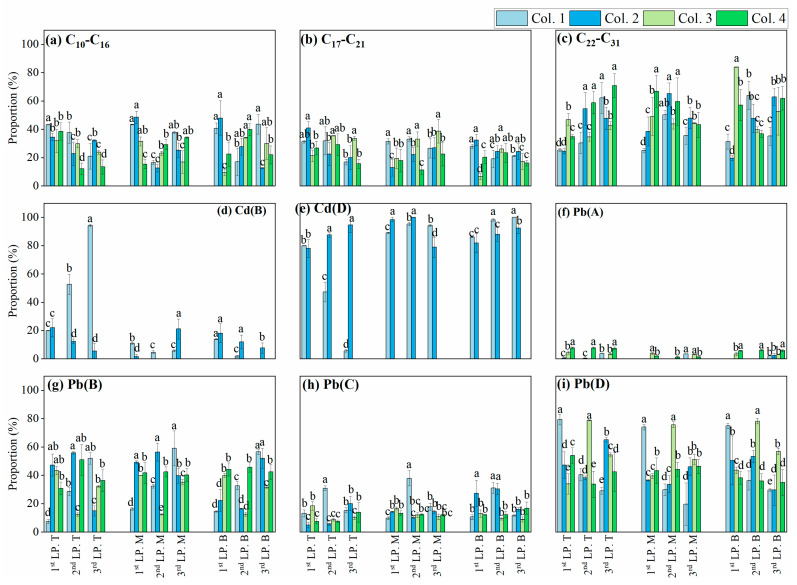
The distribution of C_10_−C_31_, Cd, and Pb in Col. 1–Col. 4 during the leaching processes. LP is an abbreviation that denotes the leaching process. The capital letters T, M, and B represent the top, middle, and bottom layers at depths of 2.0, 5.5, and 9.0 cm below the ground surface (0.0 cm). All of the data are presented as mean ± standard deviation (*n* = 3). The capital letters A, B, C, and D represent the exchangeable, reducible, oxidizable, and residual fractions of Cd/Pb. Different lowercase letters (a, b, c, and d) indicate significant differences in C_10_−C_31_/Cd/Pb levels among Col. 1–Col. 4 within the same soil layer (*p* < 0.05). Groups sharing the same letter are not significantly different.

**Figure 3:**
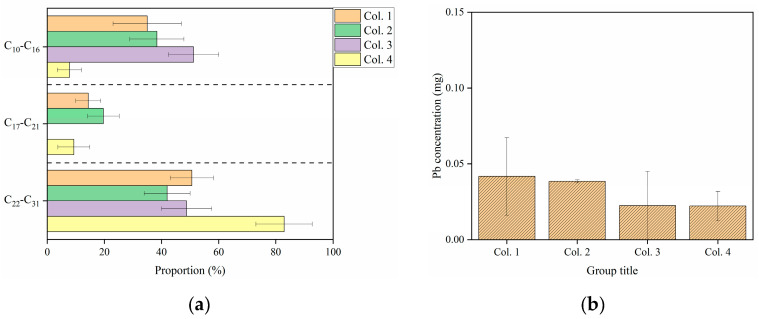
The concentrations of (**a**) C_10_−C_31_ and (**b**) Pb in the leachate. The leachate was collected only after the 3rd leaching process. Values shown are means ± standard deviation (*n* = 3). The concentration of Cd in the leachates was below detection limits and, therefore, not recorded.

During the leaching processes, the C_10_−C_16_, C_17_−C_21_, and C_22_−C_31_ fractions exhibited layer-specific accumulation (Figure 2), driven by their distinct hydrophobicity and mobility [25,42]. The distribution of these fractions in Col. 1 differed significantly from those in Col. 2 (*p* < 0.05). However, leachate analysis showed similar C_10_−C_31_ proportions between the two columns (Figure 3a). Briefly, as aging increased to 90 days, BKC showed a decrease in the C_10_−C_16_ and C_17_−C_21_ fractions but an increase in the C_22_−C_31_ fraction. This finding is consistent with previous conclusions reported for soils in non-karst areas [24,43]. On the other hand, significant differences in C_10_−C_31_ content were observed between Col. 3 and Col. 4 in both the soil layer and leachate (*p* < 0.05) (Figure 2 and Figure 3a). Specifically, extended aging led to decreased C_10_−C_16_ contents but increased C_17_−C_21_ and C_22_−C_31_ contents in BWC.

In both Col. 1 and Col. 2, Cd was predominantly present in reducible and residual fractions (Figure 2), with exchangeable and oxidizable fractions below method detection limits. Additionally, Cd concentrations in the leachate of all columns were below quantifiable limits. This fractionation pattern aligns with previous studies on soils in karst areas, where reducible and residual fractions dominate [39,44,45]. A significant difference in Cd distribution was observed between Col. 1 and Col. 2 (*p* < 0.05). Specifically, the aging process decreased the mobility of Cd in BKC. Moreover, only the residual fraction of Cd was detected in Col. 3 and Col. 4, suggesting different environmental behavior of Cd in BWC compared to BKC.

With increasing leaching frequency, the proportion of potentially bioavailable Pb (including exchangeable, reducible, and oxidizable fractions) increased in Col. 1 but showed no significant change in Col. 2. However, the concentration of Pb in the leachate of Col. 1 was similar to that in Col. 2 (Figure 3b). In this case, a significant difference in Pb distribution was observed between the two columns (*p* < 0.05), which was attributed to the aging process. Similarly, in Col. 3, the bioavailable Pb content gradually decreased during leaching, whereas in Col. 4, the content remained high (46.06–66.27%). Nevertheless, Pb concentrations in the leachate were comparable between Col. 3 and Col. 4 (Figure 3b). These observations also indicate that longer aging duration significantly decreased the high bioavailable fraction of Pb in the soil. Moreover, BKC and BWC exhibited distinct Pb transport and transformation.

### 3.2. Effects of C_10_−C_31_ in Diesel Fuel

**Figure 4:**
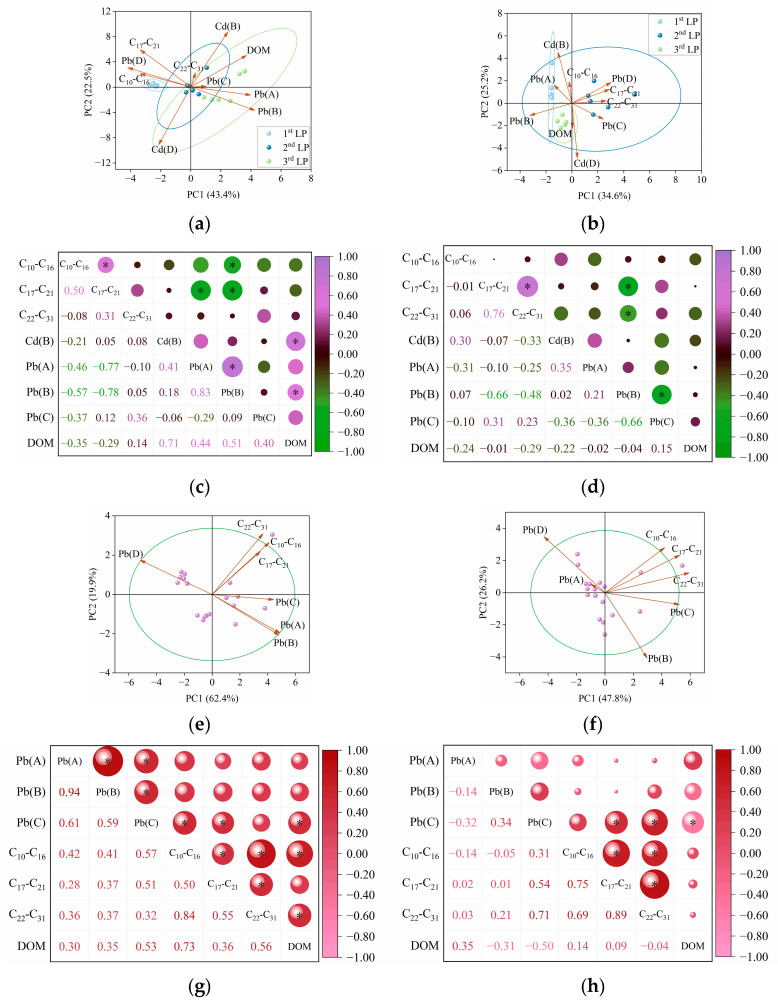
PCA and Pearson correlation analysis. (**a**,**b**) PCA for Col. 1 and Col. 2. (**c**,**d**) Pearson correlation analysis for Col. 1 and Col. 2. (**e**,**f**) PCA for Col. 3 and Col. 4. (**g**,**h**) Pearson correlation analysis for Col. 3 and Col. 4. The capital letters A, B, C, and D represent the exchangeable, reducible, oxidizable, and residual fractions of Cd/Pb. PC1 and PC2 account for the dataset. The significance of the correlations (*) is evaluated at the *p* < 0.05 level. DOM: dissolved organic matter.

**Figure 5:**
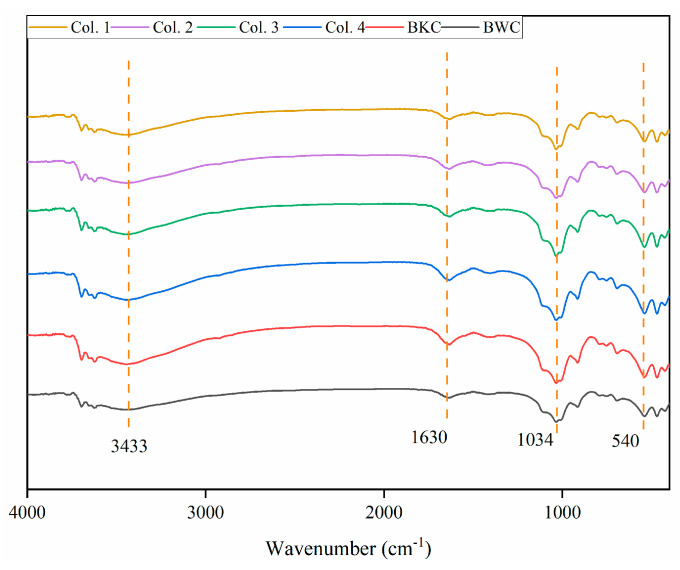
Fourier transform infrared spectroscopy (FTIR). The transmittance peaks at 3443, 1630, 1034, and 540 cm^−1^ represent the stretching of O-H, C=C/C=O, Si-O, and C=C/C-H, respectively.

In this study, diesel fuel (with a higher proportion of C_22_−C_31_, Table S4) and Cd^2+^ were initially added to BKC. Accordingly, the PCA results for Col. 1 (Figure 4a) showed that the loading value of reducible Cd closely aligned with that of C_22_−C_31_, indicating a common origin or co-introduction [8,46]. The C_10_−C_16_ fractions, characterized by high bioavailability and water solubility, were preferentially degraded and transported from the top to the bottom layer as the aging period increased from 30 to 90 days [24]. Meanwhile, the reducible fraction of Cd showed a decrease in content as the aging duration increased, particularly in the top layer. In this case, PCA results determined that C_10_−C_16_ was the dominant factor controlling the transformation of reducible Cd in Col. 2 (Figure 4b). Furthermore, an extended aging period decreased the zeta potential, increased the cation exchange capacity (CEC), and reduced the pH in Col. 1 (Table 2), consistent with previous studies [14,19]. Soils amended with diesel fuel alone also exhibited similar trends in zeta potential, CEC, and pH (Table S5). These findings suggest that changes in C_10_−C_31_ composition during natural attenuation modify soil properties. Ultimately, these modifications enhanced the electrostatic adsorption and transformation of Cd [47,48,49]. Notably, the degradation of diesel fuel may produce intermediates potentially containing Cd-binding functional groups [50,51,52,53]. However, in this study, the alteration of C_10_−C_31_ fractions in diesel fuel induced only minimal stretching of functional group intensity (including O-H, C=C/C=O, Si-O, and C=C/C-H) (Figure 5), indicating that such degradation has limited effects on the binding between Cd and soil particles. Consequently, the positive correlation between C_10_−C_16_ and reducible Cd was statistically non-significant (coefficient (*r*) = 0.30, *p* > 0.05) (Figure 4d).

Compared to Col. 3, Col. 4 showed decreased zeta potential, reduced CEC, and increased pH (Table 2 and Table S5), suggesting that aging-induced alterations in C_10_−C_31_ fractions of diesel fuel modified soil properties. Although these changes occurred, Cd primarily remained in its residual fraction. Particularly, negligible functional group changes after 90-day diesel coating (Figure 5) suggest that intermediate products of diesel fuel have little effect on Cd–soil binding. In summary, C_10_−C_31_ aging slightly influenced the transport and transformation of Cd in BWC. Instead, other characteristics of BWC potentially governed the environmental behavior of Cd.

**Figure 6:**
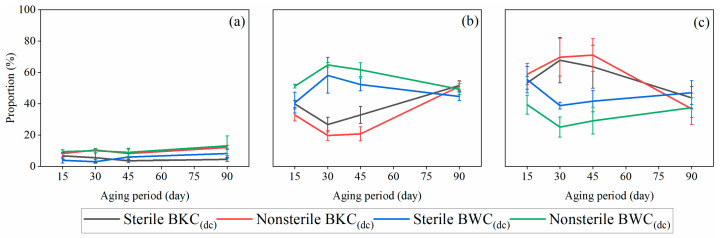
The (**a**) exchangeable, (**b**) reducible, and (**c**) oxidizable fractions of Pb in BKC_(dc)_ and BWC_(dc)_. BKC_(dc)_: BKC with an initial content of 4500 mg/kg diesel fuel and 20 mg/kg Cd. BWC_(dc)_: BWC with an initial content of 4500 mg/kg diesel fuel and 20 mg/kg Cd.

With decreasing C_10_−C_16_ content in Col. 1, the residual fraction of Pb transferred into its bioavailable fractions. In this case, the loading value of C_10_−C_16_ was closer to that of residual Pb in Col. 1 (Figure 4a). As aging progressed to 90 days, the highly bioavailable C_10_−C_16_ fractions were preferentially degraded, resulting in C_1_−C_21_ and C_22_−C_31_ becoming the dominant components in diesel fuel. Concurrently, bioavailable Pb levels decreased significantly during this period. In this case, C_17_−C_21_ and C_22_−C_31_ showed a significant negative correlation with bioavailable Pb in Col. 2 (*p* < 0.05. Figure 4c). Similarly, as the aging duration increased to 90 days, higher proportions of C_17_−C_21_ and C_22_−C_31_ fractions were observed in BWC. Subsequently, the content of oxidizable Pb showed positive correlations with C_10_−C_21_ in Col. 3 (*r* = 0.51–0.57, *p* < 0.05) and with C_17_−C_31_ in Col. 4 (*r* = 0.54–0.71, *p* < 0.05). The mechanisms underlying the interactions between Pb and C_10_−C_31_ are as follows:

As shown in the Supplementary Material (Figure S1 and Text S4), *Proteobacteria*, *Actinobacteria*, and *Firmicutes* were the dominant population in BKC and BWC. These phyla possess the ability to transform heavy metal forms and degrade PHCs [5,53]. As shown in Figure 6, significant differences in the content of reducible and oxidizable Pb were observed between sterile and nonsterile BKC_(dc)_. Similar trends were found in BWC_(dc)_. These findings indicate that the microbial degradation of diesel fuel interfered with Pb transformation in calcareous soils. Moreover, the effects of diesel degradation on Pb transformation varied between BKC and BWC, primarily due to differences in initial Pb concentrations (Table S1).

A linear relationship between the variables was assumed in the present work as a simple initial approach. The validity of this assumption was further evaluated based on the results. Subsequently, based on the retention content of C_10_−C_31_, MLR models were developed to describe the transport of Cd and Pb (Table 3). In this case, components demonstrating no significant interaction in the aforementioned analysis were excluded from the records. As shown in Table 3, the R^2^ value of the MLR models was lower in most cases. The poor fitting of Cd transport was attributed to the binding of Cd to calcium carbonate (CaCO_3_) in calcareous soils [54,55,56]. The poor fitting of Pb transport likely resulted from the nonlinear kinetics of diesel fuel biodegradation. Furthermore, the dissolved organic matter (DOM) content in BKC influenced the retention of reducible Cd, while the DOM content in BWC affected the concentrations of oxidizable Pb, C_10_−C_16_, and C_22_−C_31_ (Figure 4). These DOM-mediated interactions likely reduced the fitting efficiency of these models [57,58,59]. Particularly, the modeling efficiency for Pb transport in BWC was less satisfactory compared to BKC, likely due to the higher sensitivity of Pb to DOM in BWC (Table S1).

### 3.3. Retention of Cd and Pb in C_16_-Contaminated Soils

In this study, C_16_ was selected as representative of residual diesel in soil after long-term natural attenuation of diesel fuel. As the aging duration increased to 90 days, the concentration of C_16_ declined in the surface layer (−0.4 to 0.0 cm) of Col. 5–Col. 8 (Table S6), with a concurrent increase in C_10_−C_15_ concentrations, indicating their potential role as intermediate degradation products.

**Figure 7:**
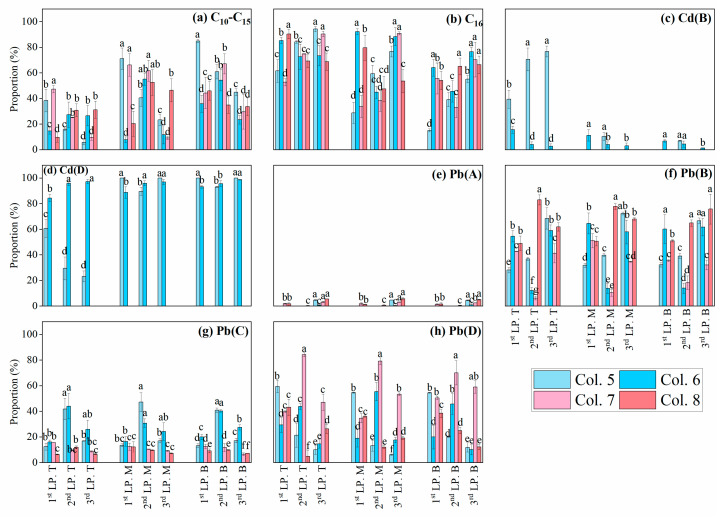
The vertical retention of C_10_−C_16_, Cd, and Pb in the column during leaching processes. LP is an abbreviation that denotes the leaching process. The capital letters T, M, and B represent the top, middle, and bottom layers at depths of 2.0, 5.5, and 9.0 cm below the ground surface, respectively. The capital letters A, B, C, and D represent the exchangeable, reducible, oxidizable, and residual fractions of Cd/Pb. All of the data are presented as mean ± standard deviation (*n* = 3). Different lowercase letters (a, b, c, and d) indicate significant differences in C_10_−C_16_/Cd/Pb levels among Col. 5–Col. 8 within the same soil layer (*p* < 0.05). Groups sharing the same letter are not significantly different.

**Figure 8:**
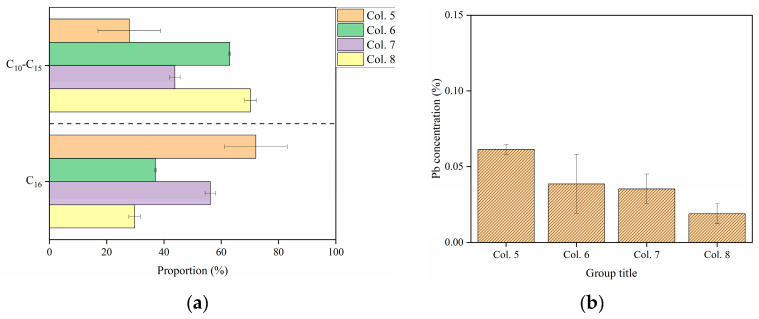
The content of (**a**) C_10_−C_16_ and (**b**) Pb in the leachate. The leachate was collected only after the 3rd leaching process. The values shown are means ± standard deviation (*n* = 3). The concentration of Cd in the leachates was below detection limits and, therefore, not recorded.

During leaching processes, C_16_ accumulated predominantly in the top layer of Col. 5, while C_10_−C_15_ preferentially migrated to the bottom layer (Figure 7), attributed to their higher mobility [25,42]. As the leaching process increased, the proportion of C_10_−C_15_ in the bottom layer of Col. 5 decreased sharply from 86.8% to 41.2%, compared to a smaller reduction from 35.88% to 23.49% in Col. 6. Consistently, Col. 5 released less C_10_−C_15_ into the leachate than Col. 6 (Figure 8a). The retention of C_10_−C_16_ was markedly different between BKC and BWC. Higher levels of C_10_−C_15_ accumulated in the middle and bottom layers of Col. 7, showing a significant difference from Col. 5. Compared to Col. 7, Col. 8 showed reduced C_10_−C_15_ concentrations in both the soil layer and leachate (Figure 7 and Figure 8a). In summary, the 90-day aging process reduced the content of C_10_−C_15_, consistent with reported patterns of PHC degradation during aging in soils from non-karst areas [60,61,62].

In both Col. 5 and Col. 6, Cd was predominantly present in reducible and residual fractions (Figure 7). The reducible fraction of Cd accumulated to high levels in the top layer of Col. 5, peaking at 76.80% in the final leaching period. In contrast, Col. 6 exhibited a lower range (12.27–15.60%) in each layer during the leaching process. In addition, no detectable Cd was observed in leachate from either column. These results indicate that the 90-day aging drove the transformation of reducible Cd into the residual fraction. Moreover, only residual Cd was measured in both Col. 7 and Col. 8. This implies distinct transformation efficiency of Cd in BWC compared to BKC.

During leaching periods, the proportion of potential bioavailable Pb increased significantly in Col. 5, whereas Col. 6 showed a lower proportion. Particularly, both the exchangeable Pb content in soil layers and the Pb concentrations in the leachate were dramatically higher in Col. 5 than Col. 6 (*p* < 0.05, Figure 7 and Figure 8b). Furthermore, Col. 7 contained 15.71–65.35% of bioavailable Pb, lower than the 56.92–95.14% range in Col. 8. In summary, the aging duration and soil type influenced the transport and transformation of Pb in soils.

### 3.4. Effects of C_16_

**Figure 9:**
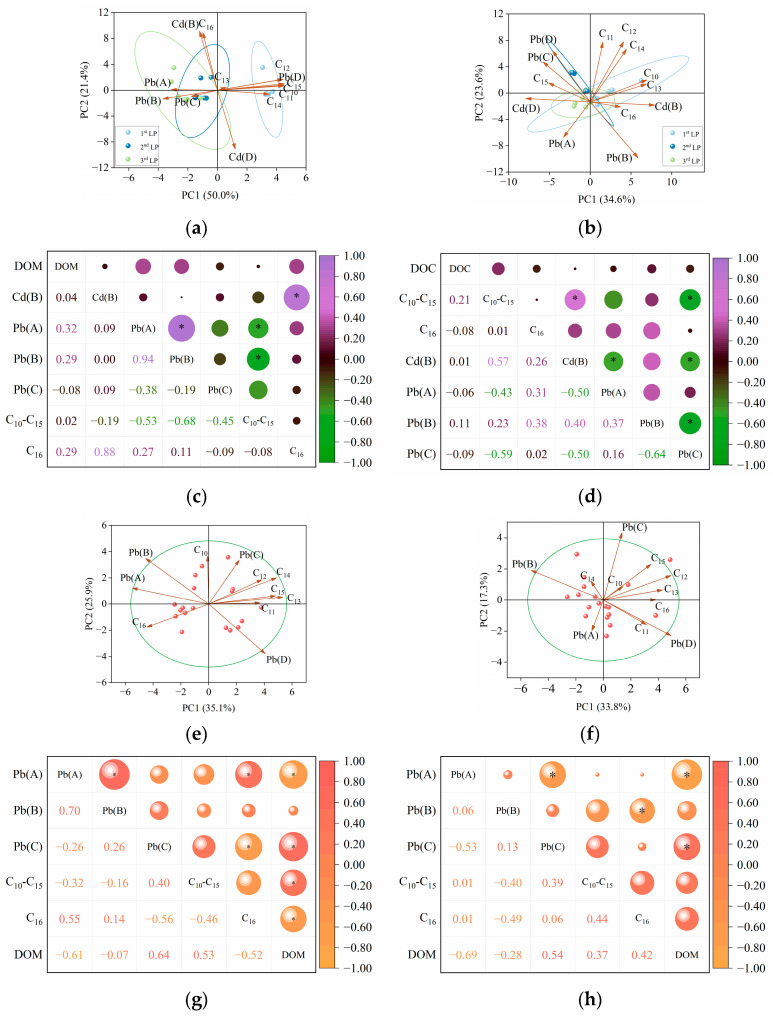
PCA and Pearson correlation analysis. (**a**,**b**) PCA for Col. 5 and Col. 6. (**c**,**d**) Pearson correlation analysis for Col. 5 and Col. 6. (**e**,**f**) PCA for Col. 7 and Col. 8. (**g**,**h**) Pearson correlation analysis for Col. 7 and Col. 8. The capital letters A, B, C, and D represent the exchangeable, reducible, oxidizable, and residual fractions of Cd/Pb. PC1 and PC2 account for the dataset. The significance of the correlations (*) is evaluated at the *p* < 0.05 level.

**Figure 10:**
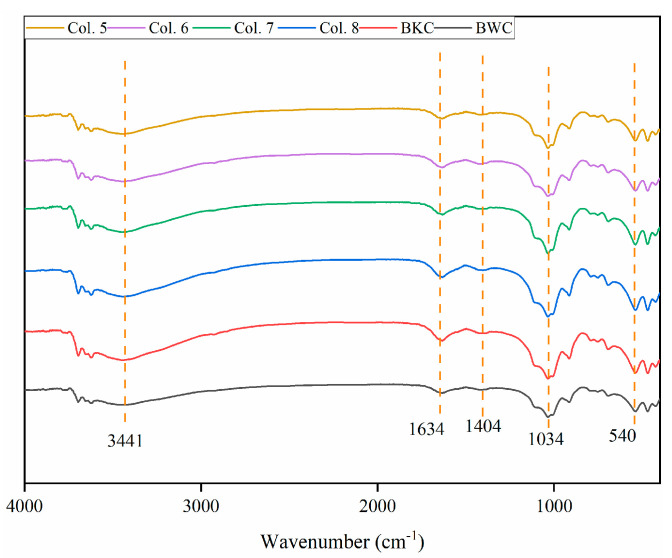
FTIR. The transmittance peaks at 3441, 1634, 1404, 1034, and 540 cm^−1^ represent the stretching of O-H, C=C/C=O, Si-O, C=C, and C=C/C-H, respectively.

The PCA results for Col. 5 (Figure 9a) explained 71.4% of the total variance in the first two principal components (PC1 and PC2), indicating a satisfactory analysis [63]. In PC1 and PC2, reducible Cd and C_16_ exhibited similar loading values, with a small angle between them. Col. 6 also showed the similar PCA results (Figure 9b). These observations suggest that Cd and C_16_ share common sources and exhibit similar behavior [64,65]. Furthermore, the Pearson correlation analysis revealed a significant positive correlation between C_16_ and reducible Cd in Col. 5 (*r* = 0.88, *p* < 0.05) (Figure 9c). Our previous work demonstrated the interaction between the reducible fraction of Cd and C_16_ using batch experiments [66]. Compared with Col. 5, Col. 6 showed decreased zeta potential, increased CEC, and elevated pH (Table 4). Similar trends in these parameters were observed in BKC coated with C_16_ alone (Table S7), suggesting that C_16_ aging drives soil property modifications. Additionally, the functional groups showed minimal peak shifts during C_16_ aging (Figure 10), suggesting that this aging process only marginally affected the interaction between Cd and soils. It is noteworthy that the humic fractions in DOM increased as the aging period increased (Figure S2). This process may result in decreased C_16_ transport, enhanced C_16_ degradation, and increased Cd complexation [27,67,68,69], ultimately reducing interactions between Cd and C_16_ in Col. 6. Consequently, Col. 6 showed only a weak positive correlation between these variables (*r* = 0.26, *p* > 0.05) (Figure 9d). Moreover, despite changes in zeta potential, CEC, and pH in Col. 7 with extended aging to 90 days (Table 4), Cd primarily remained in the residual fraction. This suggests that other physicochemical characteristics of BWC potentially governed the environmental behavior of Cd, rather than C_16_ aging.

During the aging period, the residual C_16_ became the dominant component in Col. 6, accompanied by a decrease in the content of residual Pb. In this case, a significant negative correlation was observed between bioavailable Pb content and the retention of C_10_−C_15_ in Col. 5, while this correlation decreased in Col. 6 (Figure 9c,d). On the other hand, as C_16_ became the dominant component in Col. 7, the retention of exchangeable Pb significantly increased (*r* = 0.55, *p* < 0.05) (Figure 9g). In contrast, C_16_ was negatively correlated with reducible Pb in Col. 8 (*r* = −0.49, *p* < 0.05) (Figure 9h). The mechanisms underlying the interactions between Pb and C_10_−C_16_ are as follows: As described in Section 3.2, microbial degradation of diesel fuel altered the chemical forms of Pb in the soils. Due to its simpler molecular structure, C_16_ exhibited more efficient degradation among diesel components. Therefore, C_16_ degradation potentially played a key role in governing Pb behavior in BKC and BWC. On the other hand, as C_16_ degradation progressively consumed DO in the soil environment during aging [14], the resulting oxygen limitation modulated the transformation efficiency of Pb from residual to bioavailable forms by altering microbial activity or redox conditions [70]. Moreover, the different Pb forms and microorganisms between BKC and BWC likely contributed to the distinct interactions observed between C_10_−C_16_ and Pb (Table S1 and Figure S1).

In Table 5, components demonstrating no significant interaction in the aforementioned analysis were excluded from the records. Low R^2^ values were observed in the MLR models for Cd and Pb transport in Col. 5–Col. 8 (Table 5). Specifically, the transport of reducible Cd in Col. 6 was poorly fitted by MLR models, likely due to Cd–DOM complexation and the degradation of C_10_−C_16_ during 90-day aging [27,70,71]. Additionally, Pb transport modeling was hindered by nonlinear kinetics of C_16_ degradation. DOM content further affected C_10_−C_16_ and Pb retention (Figure 9), reducing model performance.

In summary, the degradation of low-molecular-weight PHC intermediates during long-term aging led to two key transformations: (1) Cd shifted from the reducible to the residual fraction, reducing its mobility, and (2) the residual fraction of Pb became increasingly bioavailable in calcareous soils from karst regions. These findings have important environmental and practical implications. Specifically, the strong immobilization of Cd^2^⁺ suggests limited long-range transport, indicating that microbial remediation should be concentrated near the pollution source rather than across broader areas. In contrast, the enhanced bioavailability of Pb raises concerns about toxicity-induced inhibition of microbial activity, potentially limiting the effectiveness of bioremediation in calcareous karst soils. Therefore, site-specific amendments—such as biochar, chelating agents, or pH regulators—may be necessary to reduce Pb toxicity.

## 4. Conclusions

This study investigated the transport and transformation of Cd and Pb in two typical calcareous soils from karst areas, focusing on the effects of diesel fuel degradation. Key findings revealed that the low-molecular-weight fractions of diesel fuel (C_10_−C_16_) exhibited a significant decline in calcareous soils during the 90-day aging period, attributable to their high bioavailability. This degradation process altered the zeta potential, CEC, and pH of calcareous soils, which subsequently enhanced Cd retention through electrostatic attraction and drove Cd transformation from the reducible to the residual fraction. Concurrently, the alteration of the C_10_−C_16_ fraction in diesel fuel induced the transformation of Pb from residual to bioavailable forms by stimulating microbial activity. To clarify the effects of C_10_−C_16_ and minimize interference from other diesel components, calcareous soils contaminated solely with C_10_−C_16_ were also examined. The degradation of C_10_−C_16_ fractions in these soils induced Cd stabilization and Pb activation, showing patterns similar to those in diesel-contaminated calcareous soils. This consistency highlights the dominant role of C_10_−C_16_ in controlling metal behavior. Moreover, DOM and CaCO_3_ content in calcareous soils was found to significantly mediate Cd and Pb transformation, thereby weakening the interactions between these metals and C_10_−C_16_ fractions.

## Figures and Tables

**Table 1:** Experimental groups.

Group Number	Pollutants in the Surface Soils of the Column (−0.4 cm~0.0 cm)	Uncontaminated Soils (Filled from −10 cm to −0.4 cm)	Aging Periods for Contaminated Soils in the Surface Layer of the Column (−0.4 cm~0.0 cm)
Col. 1	4500 mg/kg diesel fuel + 20 mg/kg Cd	BKC	30 days
Col. 2			90 days
Col. 3		BWC	30 days
Col. 4			90 days
Col. 5	4500 mg/kg n-hexadecane (abbreviated as C_16_) + 20 mg/kg Cd	BKC	30 days
Col. 6			90 days
Col. 7		BWC	30 days
Col. 8			90 days

**Table 2:** Zeta potential and CEC in soil samples from Col. 1 to Col. 4.

	Col. 1	Col. 2	Col. 3	Col. 4	BKC	BWC
Zeta potential (mV)	−12.30	−17.22	−11.91	−15.13	−20.81	−13.58
CEC (cmol^+^/kg)	57.20	70.71	13.34	8.31	86.91	7.55
pH	8.10	8.00	5.81	6.00	8.10	5.35

The results are presented as average values (*n* = 3).

**Table 3:** MLR models for Cd and Pb transport in Col. 1–Col. 4.

Group	Equation
Col. 1	$Y_{\mathrm{Pb}(A)}=5.879-0.014X_{C_{10}-C_{16}}-0.320X_{C_{17}-C_{21}}+0.007X_{C_{22}-C_{31}}$(R^2^ = 0.53) $Y_{\mathrm{Pb}(B)}=101.242-0.453X_{C_{10}-C_{16}}-3.844X_{C_{17}-C_{21}}+0.180X_{C_{22}-C_{31}}$(R^2^ = 0.71)
Col. 2	$Y_{\mathrm{Cd}(B)}=-0.206+0.056X_{C_{10}-C_{16}}+0.093X_{C_{17}-C_{21}}-0.014X_{C_{22}-C_{31}}$(R^2^ = 0.31) $Y_{\mathrm{Pb}(B)}=87.598+0.174X_{C_{10}-C_{16}}-2.208X_{C_{17}-C_{21}}+0.016X_{C_{22}-C_{31}}$(R^2^ = 0.45)
Col. 3	$Y_{\mathrm{Pb}(C)}=5.771+0.231X_{C_{10}-C_{16}}+0.215X_{C_{17}-C_{21}}-0.023X_{C_{22}-C_{31}}$(R^2^ = 0.54)
Col. 4	$Y_{\mathrm{Pb}(C)}=16.868+0.025X_{C_{10}-C_{16}}-0.461X_{C_{17}-C_{21}}+0.026X_{C_{22}-C_{31}}$(R^2^ = 0.64)

$Y_{\mathrm{Cd}(B)}$, $Y_{\mathrm{Pb}(A)}$, $Y_{\mathrm{Pb}(B)}$, and $Y_{\mathrm{Pb}(C)}$ represent dependent variables. $X_{C_{10}-C_{16}}$, $X_{C_{17}-C_{21}}$, and $X_{C_{22}-C_{31}}$ represent independent variables.

**Table 4:** Zeta potential and CEC in soil samples from Col. 5 to Col. 8.

	Col. 5	Col. 6	Col. 7	Col. 8	BKC	BWC
Zeta potential (mV)	−13.5	−15.8	−9.11	−14.55	−20.81	−13.58
CEC (cmol^+^/kg)	45.8	61.3	12.69	8.83	86.91	7.55
pH	7.91	8.00	5.70	6.00	8.10	5.35

The results are presented as average values (*n* = 3).

**Table 5:** MLR models for Cd and Pb transport in Col. 5–Col. 8.

Group	Equation
Col. 5	$Y_{\mathrm{Cd}(B)}=0.728-0.119X_{C_{10}-C_{15}}+0.219X_{C_{16}}$(R^2^ = 0.78) $Y_{\mathrm{Pb}(A)}=3.002-0.186X_{C_{10}-C_{15}}+0.020X_{C_{16}}$(R^2^ = 0.33) $Y_{\mathrm{Pb}(B)}=67.838-1.921X_{C_{10}-C_{15}}+0.039X_{C_{16}}$(R^2^ = 0.46)
Col. 6	$Y_{\mathrm{Pb}(C)}=24.319+1.299X_{C_{10}-C_{15}}-0.081X_{C_{16}}$(R^2^ = 0.13)
Col. 7	$Y_{\mathrm{Pb}(A)}=1.128-0.038X_{C_{10}-C_{15}}+0.027X_{C_{16}}$(R^2^ = 0.31) $Y_{\mathrm{Pb}(C)}=10.183+0.194X_{C_{10}-C_{15}}-0.060X_{C_{16}}$(R^2^ = 0.34)
Col. 8	$Y_{\mathrm{Pb}(B)}=74.810-1.489X_{C_{10}-C_{15}}-0.165X_{C_{16}}$(R^2^ = 0.28)

$Y_{\mathrm{Cd}(B)}$, $Y_{\mathrm{Pb}(A)}$, $Y_{\mathrm{Pb}(B)}$, and $Y_{\mathrm{Pb}(C)}$ represent dependent variables. $X_{C_{10}-C_{15}}$ and $X_{C_{16}}$ represent independent variables selected based on the results of PCA and Pearson correlation analysis.

## Data Availability

Data are available by contacting the authors.

## Supplementary Materials

The following supporting information can be downloaded at: https://www.mdpi.com/article/10.3390/toxics13060496/s1. Additional details of soil properties are provided in the Supplementary Materials [72,73,74,75,76,77,78,79,80]. Text S1. Methodologies for determining soil properties. Text S2. Methods for extraction and analysis of C10−C31 in the samples. Text S3. Methodologies for extraction and analysis of Cd and Pb in the samples. Text S4. Analytical methods for Cd and Pb speciation in sterilized contaminated soils. Table S1. The properties of BKC and BEC. Table S2. The physicochemical characteristics of diesel fuel and n-hexadecane. Table S3. The details of the packed column. Table S4. The proportion of C10−C31 in the surface contaminated layer. Table S5. Zeta potential, CEC, and pH in Col. 1*-Col. 4*. Table S6. The proportion of C16 in the surface contaminated layer. Table S7. Zeta potential, CEC, and pH in Col. 5*-Col. 8*. Figure S1. The compositions at phyla level of microbial communities in BKC and BWC. Figure S2. The DOM fractions of soil samples.

## References

[1] Cui Y., Bai L., Li C., He Z., Liu X. (2022). Assessment of heavy metal contamination levels and health risks in environmental media in the northeast region. Sustain. Cities Soc..

[2] Wang X., Li D., Gao P., Gu W., He X., Yang W., Tang W. (2020). Analysis of biosorption and biotransformation mechanism of Pseudomonas chengduensis strain MBR under Cd(II) stress from genomic perspective. Ecotoxicol. Environ. Saf..

[3] Tan J., Yi H., Zhang Z., Meng D., Li Y., Xia L., Song S., Wu L., Sáncheze R.M.T., Farías M.E. (2022). Montmorillonite facilitated Pb(II) biomineralization by Chlorella sorokiniana FK in soil. J. Hazard. Mater..

[4] Ding C., Ding Z., Liu Q., Liu W., Chai L. (2024). Advances in mechanism for the microbial transformation of heavy metals: Implications for bioremediation strategies. Chem. Commun..

[5] Liu S.H., Zeng G.M., Niu Q.Y., Liu Y., Zhou L., Jiang L.H., Tan X.F., Xu P., Zhang C., Cheng M. (2017). Bioremediation mechanisms of combined pollution of PAHs and heavy metals by bacteria and fungi: A mini review. Bioresour. Technol..

[6] Zhou Y., Gu T., Yi W., Zhang T., Zhang Y. (2019). The release mechanism of heavy metals from lab-scale vertical flow constructed wetlands treating road runoff. Environ. Sci. Pollut. Res..

[7] Albert H.A., Li X., Jeyakumar P., Wei L., Huang L., Huang Q., Kamran M., Shaheen S.M., Hou D., Rinklebe J. (2021). Influence of biochar and soil properties on soil and plant tissue concentrations of Cd and Pb: A meta-analysis. Sci. Total Environ..

[8] Khudur L.S., Gleeson D.B., Ryan M.H., Shahsavari E., Haleyur N., Nugegoda D., Ball A.S. (2018). Implications of co-contamination with aged heavy metals and total petroleum hydrocarbons on natural attenuation and ecotoxicity in Australian soils. Environ. Pollut..

[9] Zhang J., Dai J., Chen H., Du X., Wang W., Wang R. (2012). Petroleum contamination in groundwater/air and its effects on farmland soil in the outskirt of an industrial city in China. J. Geochem. Explor..

[10] Li W., Shen Z., Tian T., Liu R., Qiu J. (2012). Temporal variation of heavy metal pollution in urban stormwater runoff. Front. Environ. Sci. Eng..

[11] Hong M., Zhang L., Tan Z., Huang Q. (2019). Effect mechanism of biochar’s zeta potential on farmland soil’s cadmium immobilization. Environ. Sci. Pollut. Res..

[12] Wang Y., Li M., Liu Z., Zhao J., Chen Y. (2021). Interactions between pyrene and heavy metals and their fates in a soil-maize (Zea mays L.) system: Perspectives from the root physiological functions and rhizosphere microbial community. Environ. Pollut..

[13] Zuo R., Shi J., Han K., Xu D., Li Q., Zhao X., Xue Z., Xu Y., Wu Z., Wang J. (2022). Response relationship of environmental factors caused by toluene concentration during leaching of capillary zone. J. Environ. Manag..

[14] Guo Y., Wen Z., Zhang C., Jakada H. (2020). Contamination and natural attenuation characteristics of petroleum hydrocarbons in a fractured karst aquifer, North China. Environ. Sci. Pollut. Res..

[15] Lv H., Su X., Wang Y., Dai Z., Liu M. (2018). Effectiveness and mechanism of natural attenuation at a petroleum-hydrocarbon contaminated site. Chemosphere.

[16] Zhang L.M., Long L.L., Zhu Q.R., Chen C., Xu M., Wu J., Yang G. (2024). Mechanism and ecological environmental risk assessment of peroxymonosulfate for the treatment of heavy metals in soil. Sci. Total Environ..

[17] Ali A., Li M., Su J., Li Y., Wang Z., Bai Y., Ali E.F., Shaheen S.M. (2022). Brevundimonas diminuta isolated from mines polluted soil immobilized cadmium (Cd^2+^) and zinc (Zn^2+^) through calcium carbonate precipitation: Microscopic and spectroscopic investigations. Sci. Total Environ..

[18] Qi S., Li X., Luo J., Han R., Chen Q., Shen D., Shentu J. (2022). Soil heterogeneity influence on the distribution of heavy metals in soil during acid rain infiltration: Experimental and numerical modeling. J. Environ. Manag..

[19] Yang W., Wang Y., Sharma P., Li B., Liu K., Liu J., Flury M., Shang J. (2017). Effect of naphthalene on transport and retention of biochar colloids through saturated porous media. Colloids Surf. A Physicochem. Eng. Asp..

[20] Fonseca B., Pazos M., Figueiredo H., Tavares T., Sanromán M.A. (2011). Desorption kinetics of phenanthrene and lead from historically contaminated soil. Chem. Eng. J..

[21] Liu X., Guo H., Zhang X., Zhang S., Cao X., Lou Z., Zhang W., Chen Z. (2022). Modeling the transport behavior of Pb(II), Ni(II) and Cd(II) in the complex heavy metal pollution site under the influence of coexisting ions. Process Saf. Environ. Prot..

[22] Liu G., Zhong H., Yang X., Liu Y., Shao B., Liu Z. (2018). Advances in applications of rhamnolipids biosurfactant in environmental remediation: A review. Biotechnol. Bioeng..

[23] Logeshwaran P., Megharaj M., Chadalavada S., Bowman M., Naidu R. (2018). Petroleum hydrocarbons (PH) in groundwater aquifers: An overview of environmental fate, toxicity, microbial degradation and risk-based remediation approaches. Environ. Technol. Innov..

[24] Li Y., Zheng B., Yang Y., Chen K., Chen X., Huang X., Wang X. (2022). Soil microbial ecological effect of shale gas oil-based drilling cuttings pyrolysis residue used as soil covering material. J. Hazard. Mater..

[25] Sun Y., Zhang S., Xie Z., Lan J., Li T., Yuan D., Yang H., Xing B. (2020). Characteristics and ecological risk assessment of polycyclic aromatic hydrocarbons in soil seepage water in karst terrains, southwest China. Ecotoxicol. Environ. Saf..

[26] Yan J., Li Q., Hu L., Wang J., Zhou Q., Zhong J. (2022). Response of microbial communities and their metabolic functions to calcareous succession process. Sci. Total Environ..

[27] Chen C.H., Liu P.W.G., Whang L.M. (2019). Effects of natural organic matters on bioavailability of petroleum hydrocarbons in soil-water environments. Chemosphere.

[28] Rajasekhar B., Nambi I.M., Govindarajan S.K. (2021). Investigating the degradation of nC_12_ to nC_23_ alkanes and PAHs in petroleum- contaminated water by electrochemical advanced oxidation process using an inexpensive Ti/Sb-SnO_2_/PbO_2_ anode. Chem. Eng. J..

[29] Xia X., Ji J., Zhang C., Yang Z., Shi H. (2022). Carbonate bedrock control of soil Cd background in Southwestern China: Its extent and influencing factors based on spatial analysis. Chemosphere.

[30] Li Y.T., Wang Y.Q., Li X., Liu X.Y., Liu H., Sui Q., Du W.Y. (2024). Remediation of petroleum hydrocarbon contaminated soils by nZVI coupled with electrokinetic activation of persulfate. J. Clean. Prod..

[31] Fu X., Cui Z., Zang G. (2014). Migration, speciation and distribution of heavy metals in an oil-polluted soil affected by crude oil extraction processes. Environ. Sci. Process. Impacts.

[32] *GB36600-2018*; Ministry of Ecology and Environment the People’s Republic of China. Soil Environmental Quality Risk Control Standard for Soil Contamination of Development Land. https://www.mee.gov.cn/ywgz/fgbz/bz/bzwb/trhj/201807/t20180703_446027.shtml.

[33] Zhang X., Huang C., Ren J., Bekele T.G., Zhao H. (2022). Effect of cyclodextrin on desorption of petroleum hydrocarbons in soil. Process Saf. Environ. Prot..

[34] Liu N., Wang L., Cao D., Li D., Zhu Y., Huang S., Shi J. (2023). Remediation of petroleum contaminated soil by persulfate oxidation coupled with microbial degradation. J. Environ. Chem. Eng..

[35] Wu M., Wu J., Zhang X., Ye X. (2019). Effect of bioaugmentation and biostimulation on hydrocarbon degradation and microbial community composition in petroleum-contaminated loessal soil. Chemosphere.

[36] Huang J., Li X., Tian Y., Sun J., Yang Q., Yang M., Wang S. (2024). A novel evaluation method-based effect analysis of urbanization on extreme precipitation in Guangxi, South China. Theor. Appl. Climatol..

[37] Cai L., Chen X., Huang R., Smettem K. (2022). Runoff change induced by vegetation recovery and climate change over carbonate and non-carbonate areas in the karst region of South-west China. J. Hydrol..

[38] Meng Q., Xing L., Liu L., Xing X., Zhao Z., Zhang F., Li C. (2021). Time-lag characteristics of the response of karst springs to precipitation in the northern China. Environ. Earth Sci..

[39] Wen Y., Li W., Yang Z., Zhang Q., Ji J. (2020). Enrichment and source identification of Cd and other heavy metals in soils with high geochemical background in the karst region, Southwestern China. Chemosphere.

[40] Park I.S., Park J.W. (2010). A novel total petroleum hydrocarbon fractionation strategy for human health risk assessment for petroleum hydrocarbon-contaminated site management. J. Hazard. Mater..

[41] Okonkwo C.J., Liu N., Li J., Ahmed A. (2022). Experimental thawing events enhance petroleum hydrocarbons attenuation and enzymatic activities in polluted temperate soils. Int. J. Environ. Sci. Technol..

[42] Cai T., Ding Y., Zhang Z., Wang X., Wang T., Ren Y., Dong Y. (2019). Effects of total organic carbon content and leaching water volume on migration behavior of polycyclic aromatic hydrocarbons in soils by column leaching tests. Environ. Pollut..

[43] Chen H., Hao Y., Zhang S.L., Pan J.R., Lang M.F., Guo X.T. (2024). Vertical migration and variation of crude oil in soil around typical oilfields under natural leaching. Int. J. Environ. Sci. Technol..

[44] Wei X., Bai X., Wen X., Liu L., Xiong J., Yang C. (2023). A large and overlooked Cd source in karst areas: The migration and origin of Cd during soil formation and erosion. Sci. Total Environ..

[45] Liu Y., Xiao T., Perkins R.B., Zhu J., Zhu Z., Xiong Y., Ning Z. (2017). Geogenic cadmium pollution and potential health risks, with emphasis on black shale. J. Geochem. Explor..

[46] Anaman R., Peng C., Jiang Z., Liu X., Zhou Z., Guo Z., Xiao X. (2022). Identifying sources and transport routes of heavy metals in soil with different land uses around a smelting site by GIS based PCA and PMF. Sci. Total Environ..

[47] Liu H., Xu F., Xie Y., Wang C., Zhang A., Li L., Xu H. (2018). Effect of modified coconut shell biochar on availability of heavy metals and biochemical characteristics of soil in multiple heavy metals contaminated soil. Sci. Total Environ..

[48] Sarkodie E.K., Jiang L., Li K., Guo Z., Yang J., Shi J., Peng Y., Wu X., Huang S., Deng Y. (2024). The influence of cysteine in transformation of Cd fractionation and microbial community structure and functional profile in contaminated paddy soil. Sci. Total Environ..

[49] Wang Y., Lu T., Zhang H., Li Y., Song Y., Chen J., Fu X., Qi Z., Zhang Q. (2020). Factors affecting the transport of petroleum colloids in saturated porous media. Colloids Surf. A Physicochem. Eng. Asp..

[50] Yang T., Xu Y., Huang Q., Sun Y., Liang X., Wang L. (2022). Removal mechanisms of Cd from water and soil using Fe–Mn oxides modified biochar. Environ. Res..

[51] Li Y., Wang K., Dötterl S., Xu J., Garland G., Liu X. (2024). The critical role of organic matter for cadmium-lead interactions in soil: Mechanisms and risks. J. Hazard. Mater..

[52] Sawalha M.F., Peralta-Videa J.R., Saupe G.B., Dokken K.M., Gardea-Torresdey J.L. (2007). Using FTIR to corroborate the identity of functional groups involved in the binding of Cd and Cr to saltbush (*Atriplex canescens*) biomass. Chemosphere.

[53] Varjani S.J. (2017). Microbial degradation of petroleum hydrocarbons. Bioresour. Technol..

[54] Yin T., Lin H., Dong Y., Li B., He Y., Liu C., Chen X. (2021). A novel constructed carbonate-mineralized functional bacterial consortium for high-efficiency cadmium biomineralization. J. Hazard. Mater..

[55] Jin C., Li Z., Huang M., Wen J., Ding X., Zhou M., Cai C. (2021). Laboratory and simulation study on the Cd(Ⅱ) adsorption by lake sediment: Mechanism and influencing factors. Environ. Res..

[56] Wang Y., Li Y., Zhang Y., Wei W. (2019). Effects of macromolecular humic/fulvic acid on Cd(II) adsorption onto reed-derived biochar as compared with tannic acid. Int. J. Biol. Macromol..

[57] Zhang X., Su C., Liu X., Liu Z., Gu P., Deng M., Liu Q. (2020). Periodical changes of dissolved organic matter (DOM) properties induced by biochar application and its impact on downward migration of heavy metals under flood conditions. J. Clean. Prod..

[58] Yu H., Huang G.H., An C.H., Wei J. (2011). Combined effects of DOM extracted from site soil/compost and biosurfactant on the sorption and desorption of PAHs in a soil–water system. J. Hazard. Mater..

[59] Bao T., Wang P., Hu B., Jin Q., Zheng T., Li D. (2024). Adsorption and distribution of heavy metals in aquatic environments: The role of colloids and effects of environmental factors. J. Hazard. Mater..

[60] Tang J., Lu X., Sun Q., Zhu W. (2012). Aging effect of petroleum hydrocarbons in soil under different attenuation conditions. Agric. Ecosyst. Environ..

[61] Charrasse B., Hennebert P., Doumenq P. (2018). Mobility of PAHs, PCBs and TPHs from Fresh and Aged Dredged Sediments. Waste Biomass Valorization.

[62] Ma L., Zhang J., Han L., Li W., Xu L., Hu F., Li H. (2012). The effects of aging time on the fraction distribution and bioavailability of PAH. Chemosphere.

[63] Frescura L.M., de Menezes B.B., Duarte R., da Rosa M.B. (2020). Application of multivariate analysis on naphthalene adsorption in aqueous solutions. Environ. Sci. Pollut. Res..

[64] Peng S. (2015). The nutrient, total petroleum hydrocarbon and heavy metal contents in the seawater of Bohai Bay, China: Temporal–spatial variations, sources, pollution statuses, and ecological risks. Mar. Pollut. Bull..

[65] Bi C., Chen Y., Zhao Z., Li Q., Zhou Q., Ye Z., Ge X. (2020). Characteristics, sources and health risks of toxic species (PCDD/Fs, PAHs and heavy metals) in PM_2.5_ during fall and winter in an industrial area. Chemosphere.

[66] Huang Y., Tang Y., Liang Y., Xie Z., Wu J., Huang J., Wei S., Nie S., Jiang T. (2023). Transport and retention of n-hexadecane in cadmium-/naphthalene-contaminated calcareous soil sampled in a karst area. Environ. Geochem. Health.

[67] Li M., Chen C., Zhang W., Cao L., Zhang X., Wang Y., Xian Q. (2024). The effects of Cu-phenanthrene co-contamination on adsorption-desorption behaviors of phenanthrene in soils. Chemosphere.

[68] Yu Y., Wan Y., Camara A.Y., Li H. (2018). Effects of the addition and aging of humic acid-based amendments on the solubility of Cd in soil solution and its accumulation in rice. Chemosphere.

[69] Xie J., Dong A., Liu J., Su J., Hu P., Xu C., Chen J., Wu Q. (2019). Relevance of dissolved organic matter generated from green manuring of Chinese milk vetch in relation to water-soluble cadmium. Environ. Sci. Pollut. Res..

[70] Kang M., Tian Y., Peng S., Wang M. (2019). Effect of dissolved oxygen and nutrient levels on heavy metal contents and fractions in river surface sediments. Sci. Total Environ..

[71] Wang C., Zhou S., Song J., Wu S. (2018). Human health risks of polycyclic aromatic hydrocarbons in the urban soils of Nanjing, China. Sci. Total Environ..

[72] *HJ 889-2017*; Ministry of Ecology and Environment the People’s Republic of China. Soil Quality-Determination of Cation Exchange Capacity(CEC)-Hexamminecobalt Trichloride Solution-Spectrophotometric Method. https://english.mee.gov.cn/Resources/standards/Soil/Method_Standard4/201801/t20180105_429211.shtml.

[73] Wang Y., Zhang X., Zhang X., Meng Q., Gao F., Zhang Y. (2017). Characterization of spectral responses of dissolved organic matter (DOM) for atrazine binding during the sorption process onto black soil. Chemosphere.

[74] *HJ 911-2017*; Ministry of Ecology and Environment the People’s Republic of China. Soil and Sediment-Extraction of Organic compounds-Ultrasonic Extraction. https://codeofchina.com/standard/HJ911-2017.html.

[75] *HJ 894-2017*; Ministry of Ecology and Environment the People’s Republic of China. Water Quality-Determination of Extractable Petroleum Hydrocarbons (C10–C40) Gas Chromatography. https://english.mee.gov.cn/Resources/standards/water_environment/method_standard2/201801/t20180105_429205.shtml.

[76] Qureshi A.A., Kazi T.G., Baig J.A., Arain M.B., Afridi H.I. (2020). Exposure of heavy metals in coal gangue soil, in and outside the mining area using BCR conventional and vortex assisted and single step extraction methods. Impact on orchard grass. Chemosphere.

[77] Zhao L., Yan Y., Yu R., Hu G., Cheng Y., Huang H. (2020). Source apportionment and health risks of the bioavailable and residual fractions of heavy metals in the park soils in a coastal city of China using a receptor model combined with Pb isotopes. CATENA.

[78] *HJ 832–2017*; Ministry of Ecology and Environment the People’s Republic of China. Soil and Sediment-Digestion of Total Metal Elements- Microwave Assisted Acid Digestion Method. https://english.mee.gov.cn/Resources/standards/Soil/Method_Standard4/201708/t20170830_420636.shtml.

[79] *HJ 678–2013*; Ministry of Ecology and Environment the People’s Republic of China. Water Quality-Digestion of Total Metals-Microwave Assisted Acid Digestion Method. https://www.codeofchina.com/standard/HJ678-2013.html.

[80] Huang Z., Chen Q., Yao Y., Chen Z., Zhou J. (2021). Micro-bubbles enhanced removal of diesel oil from the contaminated soil in washing/flushing with surfactant and additives. J. Environ. Manag..

