# Supplementary material for: Degradation of Low-Molecular-Weight Diesel Fractions (C10−C16 Alkane) Drives Cd Stabilization and Pb Activation in Calcareous Soils from Karst Areas
Source: Toxics. 2025 Jun 13;13(6):496. doi: 10.3390/toxics13060496 (PMC12197093; doi:10.3390/toxics13060496)
Supplement: Supplementary file 1 [file toxics-13-00496-s001.zip › toxics-3670328-supplementary.pdf]

Supplementary material

Degradation of Low-Molecular-Weight Diesel Fractions (C_{10}-C_{16} Alkane) Drives Cd Stabilization and Pb Activation in Calcareous Soils from Karst Areas

Yiting Huang [a] · Yankui Tang [b,*] · Zhenze Xie [b] · Jipeng Wu [b] ·

Jiajie Huang [b] · Shanxiong Wei [b]

[a] School of Civil Engineering and Architecture, Guangxi University, Nanning 530004, China

[b] School of Resources, Environment, and Materials, Guangxi University, Nanning 530004, China

* Corresponding author: Yankui Tang (E-mail: 1910403002@gxu.edu.cn)

**Title**	**Page**
Text S1 Methodologies for determining soil properties.	1
Text S2 Methods for extraction and analysis of C_{10}-C_{31} in the samples.	1
Text S3 Methodologies for extraction and analysis of Cd and Pb in the samples.	3
Text S4 Analytical methods for Cd and Pb speciation in sterilized contaminated soils.	5
Table S1 The properties of BKC and BEC.	6
Table S2 The physicochemical characteristics of diesel fuel and n-hexadecane.	6
Table S3 The details of the packed column.	6
Table S4 The proportion of C_{10}-C_{31} in the surface contaminated layer.	7
Table S5 Zeta potential, CEC, and pH in Col. 1*-Col. 4*.	7
Table S6 The proportion of C_{16} in the surface contaminated layer.	7
Table S7 Zeta potential, CEC, and pH in Col. 5*-Col. 8*.	7
Figure S1 The compositions at phyla level of microbial communities in BKC and BWC.	8
Figure S2 The DOM fractions of soil samples.	8
References for supporting information.	9

Text S1 Methodologies for determining soil properties.

A pH monitor (A211, Thermo Fisher Scientific, America) was used to measure the pH value in soil samples. The Cation Exchange Capacity (CEC) was determined using Hexamminecobalt Trichloride Solution-Spectrophotometric method in China (HJ 889-2017) [1]. Zeta potential was measured using a Zetasizer apparatus (Nano ZS90, Malvern Instruments, UK). Fourier transform infrared spectroscopy (FTIR, IRTracer-100, Shimadzu, Japan) was used to determine the functional groups in the freeze-dried samples at wave numbers from 400 to 4000 cm^{-1}.

Before determining dissolved organic matter (DOM), soil samples were pretreated following the method described in [2]. More specifically, 2 g of soil sample was added to a 100 mL conical flask containing 20 mL of deionized water and 0.01 mol/L calcium chloride ($CaCl_2$, Kemel-Shanghai). Following the 24 h shaking period at 125 rpm and 25 °C, the mixture was centrifugated at 5000 rpm for 10 min. The liquid sample served to determine the concentration of DOM by TOC-VCPH (Shimadzu, Japan).

Text S2 Methods for extraction and analysis of C_{10}-C_{31} in the samples.

An amount of 2.0 g of soil sample was mixed with 2.0 g of anhydrous sodium sulfate (Na_2SO_4. Kermel-Shanghai) to eliminate the interference of moisture on the experimental results. Then, 10 mL of n-hexane (C_6H_{14}. Fuyu-Chemical, China) was added to the dried soil for C_{10}-C_{31} extraction, followed by 5 min of ultrasound sonication. This extraction procedure was conducted in accordance with the Soil and sediment–Extraction of Organic Compounds–Ultrasonic Extraction in China (HJ 991-

2017) [3]. On the other hand, 10 mL of n-hexane was added to the leachate for C_{10}-C_{31} extraction. The extraction procedure followed the method outlined in the Chinese standard for Water quality–Determination of extractable petroleum hydrocarbons (C_{10}-C_{40})–Gas chromatography (HJ 894-2017) [4].

In this study, the extraction procedures were replicated three times to control the data quality. The extraction recovery rates of the C_{10}-C_{31} in samples were between 78.6% and 85.0%.

The concentrations of C_{10}-C_{31} in the extraction solution (n-hexane) were determined using a Gas Chromatograph (GC, Shimadzu GC-2010, Japan) equipped with a flame ionization detector (FID). Additionally, the standard n-alkanes solution containing C_{10}-C_{31}, purchased from ANPEL Laboratory Technologies (Shanghai), was used to generate the standard curve. The procedures for measuring C_{10}-C_{31} in the GC system were taken from the Determination of Extractable Petroleum Hydrocarbons (C_{10}-C_{40}) in China (HJ 894-2017) [4]. Specifically, a HP-5 MS column coated with 5% phenyl-methylpolysiloxane was utilized. The injection volume was 1.0 μL, and the temperature programs for GC analysis were as follows: (1) the initial temperature was set at 60 °C and held for 1 min; (2) the temperature was then increased up to 290 °C at a rate of 8 °C/min (and held for 2 min); and (3) finally, the temperature was further increased to 300 °C at 30 °C/min (and held for 7 min).

Text S3 Methodologies for extraction and analysis of Cd and Pb in the samples.

The BCR sequential extraction process was performed to determine the chemical fractions of Cd and Pb in soil samples [5,6]. The steps are shown as follows:

(1) Step 1. Exchangeable fraction (A).

An amount of 0.5 g of air-dried soil sample was mixed with 20 mL of 0.11 mol/L acetic acid (CH_3COOH, GHTECH-Chemical, Guangdong) in a 50 mL centrifuge tube. After shaking for 16 h at 20±1 °C, this mixture was centrifugated at 4000 rpm for 20 min. Then, the concentration of the exchangeable fraction of heavy metals in the supernatant was analyzed using an Atomic Absorption Spectrophotometer (AAS, AA-7000, Shimadzu, Japan).

(2) Step 2. Reducible fraction (B).

An amount of 20 mL of 0.5 mol/L hydroxylamine hydrochloride solution ($HONH_2HCl$, Damao-Chemical, Tianjin), with its pH adjusted to 1.5 using 1.0 mol/L of nitric acid (HNO_3, Sinopharm, China), was added into the residue from Step 1, followed by shaking for 16 h. The mixture was separated by a centrifuge at 4000 rpm for 20 min. The concentration of the reducible fraction of heavy metals in the supernatant was determined by an AAS.

(3) Step 3. Oxidizable fraction (C).

The residue from Step 2 was treated twice with 5.0 mL of 30% hydrogen peroxide (H_2O_2, Sinopharm, China), and then the mixture was heated in a water bath at 85 °C until the volume of the solution was reduced to 1-2 mL. After cooling the samples to

room temperature, 25 mL of 1.0 mol/L ammonium acetate solution (CH_3COONH_4, GHTECH-Chemical, Guangdong), with its pH adjusted to 2 using 1 mol/L HNO_3, was added to these samples. The mixture was shaken for 16 h at 20±1 ℃, and then centrifugated at 4000 rpm for 20 min. Subsequently, the concentration of the oxidizable fraction of heavy metals in the supernatant was analyzed by an AAS.

(4) Step 4. Residual fraction (D).

The residue from Step 3 was digested using a mixture of acids, including 2 mL of HNO_3, 1 mL of H_2O_2, and 0.5 mL of hydrofluoric acid (HF, Sinopharm, China). The digestion procedure followed Soil and Sediment–Digestion of Total Metal Elements–Microwave-Assisted Acid Digestion Method in China (HJ 832-2017) [7], and the standard soil (National Research Center for Reference Materials, GBW07405 (GSS-5), China) was used to calculate the recovery. Finally, the concentration of the residual fraction of heavy metals in the digestion solutions was analyzed by an AAS.

According to the Water quality–Digestion of total metals–Microwave-assisted acid digestion method in China (HJ 678-2013) [8], the leachate was initially subjected to digestion, followed by the analysis of Cd and Pb concentrations by AAS.

The total content of Cd and Pb in soil samples was measured following the Soil and Sediment–Digestion of Total Metal Elements–Microwave-Assisted Acid Digestion Method in China (HJ 832-2017) [7] and analyzed using an AAS. The recovery of heavy metals was calculated using the standard soil (National Research Center for Reference Materials, GBW07405 (GSS-5), China).

In this study, the recovery rates of Cd and Pb in the soil ranged from 83.2% to 89.3%.

Text S4 Analytical methods for Cd and Pb speciation in sterilized contaminated soils.

The incubation of sterilized contaminated soils was carried out as follows: (1) Milli-Q water, containers, soil samples (including black calcareous soils (BKC) and brown calcareous soils (BWC)), and other materials were sterilized by autoclaving. (2) A stock solution was prepared by dissolving diesel fuel and $CdCl_2$ in a mixture of acetone and sterilized Milli-Q water (1%, w/w). The solution was then stirred continuously for 24 h using a magnetic stirrer to ensure homogeneity. (3) An amount of 150 mL of the stock solution was added to 1.0 kg of sterilized BKC with continuous stirring, achieving concentrations of 20 mg/kg Cd and 4500 mg/kg diesel fuel in the soil. The same procedure was repeated for sterilized BWC. (4) All contaminated soils were aged in the dark at 25 °C, with soil moisture maintained at 15 % (w/w) by weekly replenishment with sterilized Milli-Q water. (5) After aging for 15, 30, 45, and 90 days, soil samples were collected, and the chemical forms of Cd and Pb in the samples were determined using the methodology described in S3. Moreover, the microbial composition analysis was outsourced to Sangon Biotech (Shanghai) Co., Ltd, a reputable company with multiple international branches and a strong track record in molecular biology services.

Table S1 The properties of BKC and BWC.

Soils	Total Cd (mg/kg)	Total Pb (mg/kg)	C_{10}-C_{31} (mg/kg)	DOM (mg/kg)
BKC	8.5	124.2	-	129.4
BWC	0.51	95.65	-	36.88

The results are presented as average values (n=3). A dash (-) indicates that the value was below the detection limit. BKC: black calcareous soils. BWC: brown calcareous soils.

Table S2 The physicochemical characteristics of diesel fuel and n-hexadecane.

Reagents	Log K_{ow}[b] (25°C)	Density (25°C) (g/cm^3)	Surface tension (mN/m)	Dynamic viscosity (25°C) (mPa s)	Molecular mass (g/mol)	Water-solubility (25°C) (mg/L)	Volatility (25°C) (mm Hg)
Diesel fuel	*	0.85	17.12	3.03	230.89 [c]	*	*
N-hexadecane ($C_{16}H_{34}$) [a]	6.70	0.77	27.05	3.03	226.44	2.1×10^{-5}	1.49×10^{-3}

[a] N-hexadecane is abbreviated as C_{16}. CAS: 544-76-3. The n-hexadecane reagent used in this study had a purity of 98%. [b] Octanol–Water Partition Coefficient. [c] This value was averaged, as reported by [9]. The '*' indicates that the value varies depending on the fractions of petroleum hydrocarbons (PHCs) and the experimental conditions.

Table S3 The details of the packed column.

Group title	Weight of the uncontaminated soils (g)	Height of uncontaminated layer (cm)	Weight of the contaminated soils (g)	Height of contaminated layer (cm)	The total weight of soils (g)
Col. 1	285.0±0.1	-9.9~-0.4	30.0±0.1	-0.4~-0.0	315.0±0.1
Col. 2	285.0±0.1	-10.1~-0.4	30.0±0.1	-0.4~-0.0	315.0±0.1
Col. 3	285.0±0.1	-10.0~-0.4	30.0±0.1	-0.4~-0.0	315.0±0.1
Col. 4	285.0±0.1	-10.0~-0.4	30.0±0.1	-0.4~-0.0	315.0±0.1
Col. 5	285.0±0.1	-10.1~-0.4	30.0±0.1	-0.4~-0.0	315.0±0.1
Col. 6	285.0±0.1	-10.1~-0.4	30.0±0.1	-0.4~-0.0	315.0±0.1
Col. 7	285.0±0.1	-10.0~-0.4	30.0±0.1	-0.4~-0.0	315.0±0.1
Col. 8	285.0±0.1	-10.0~-0.4	30.0±0.1	-0.4~-0.0	315.0±0.1

The values in the table were averaged (n=3). The surface of the ground is designated as 0.0 cm, with negative values indicating depths below the ground surface.

Col.1: BKC with initial concentrations of 4500 mg/kg diesel fuel and 20 mg/kg Cd, aged for 30 days. Col. 2: BKC with initial concentrations of 4500 mg/kg diesel fuel and 20 mg/kg Cd, aged for 90 days. Col. 3: BWC with initial concentrations of 4500 mg/kg diesel fuel and 20 mg/kg Cd, aged for 30 days. Col. 4: BWC with initial concentrations of 4500 mg/kg diesel fuel and 20 mg/kg Cd, aged for 90 days. Col.5: BKC with initial concentrations of 4500 mg/kg C_{16} and 20 mg/kg Cd, aged for 30 days. Col. 6: BKC with initial concentrations of 4500 mg/kg C_{16} and 20 mg/kg Cd, aged for 90 days. Col. 7: BWC with initial concentrations of 4500 mg/kg C_{16} and 20 mg/kg Cd, aged for 30 days. Col. 8: BWC with initial concentrations of 4500 mg/kg C_{16} and 20 mg/kg Cd, aged for 90 days.

Table S4 The proportion of C_{10}-C_{31} in the surface contaminated layer (-0.4 cm-0.0 cm).

Group number	C_{10}-C_{16} (%)	C_{17}-C_{21} (%)	C_{22}-C_{31} (%)
Col. 1	13.87	24.72	61.40
Col. 2	16.19	20.98	62.83
Col. 3	10.96	15.44	73.60
Col. 4	12.33	17.51	70.16

At day 0 of aging, C_{10}-C_{16}, C_{17}-C_{21}, and C_{22}-C_{31} concentrations in diesel-contaminated BKC were 8.27%, 9.64%, and 82.09%, respectively, while C_{10}-C_{16}, C_{17}-C_{21}, and C_{22}-C_{31} in diesel-contaminated BWC were 3.35%, 9.78%, and 86.87%, respectively.

Table S5 Zeta potential, CEC, and pH in Col. 1*-Col. 4*.

	Col. 1*	Col. 2*	Col. 3*	Col. 4*
Zeta potential (mV)	-16.4	-19.5	-15.88	-16.94
CEC ($cmol^{+}$/kg)	86.9	78.5	6.58	8.37
pH	8.1	8.2	5.40	5.13

The results are presented as average values (n=3). Col. 1*-Col. 4*: Col. 1-Col. 4 without Cd addition.

Table S6 The proportion of C_{16} in the surface contaminated layer.

Group number	Col. 5	Col. 6	Col. 7	Col. 8
C_{16} content (mg/kg)	4133.0	3743.3	3822.31	3106.82

The surface contaminated layer: -0.4 cm-0.0 cm.

Initially (day 0 of aging), the C_{16} concentrations in contaminated BKC and BWC were 4501.4 mg/kg and 4510.6 mg/kg, respectively.

Table S7 Zeta potential, CEC, and pH in Col. 5*-Col. 8*.

	Col. 5*	Col. 6*	Col. 7*	Col. 8*
Zeta potential (mV)	-15.2	-17.3	-12.57	-14.38
CEC ($cmol^{+}$/kg)	72.6	88.7	8.69	10.98
pH	8.1	7.8	5.46	5.88

The results are presented as average values (n=3). Col. 5*-Col. 8*: Col. 5-Col. 8 without Cd addition.

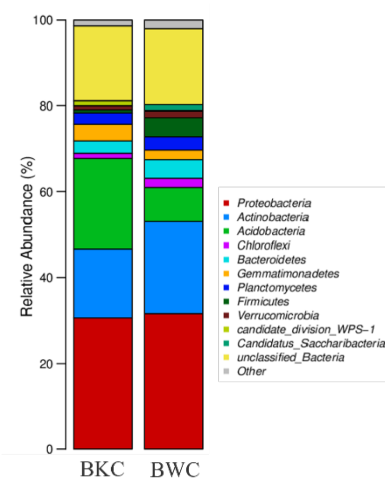

Figure S1. The compositions at the phyla level of microbial communities in BKC and BWC.

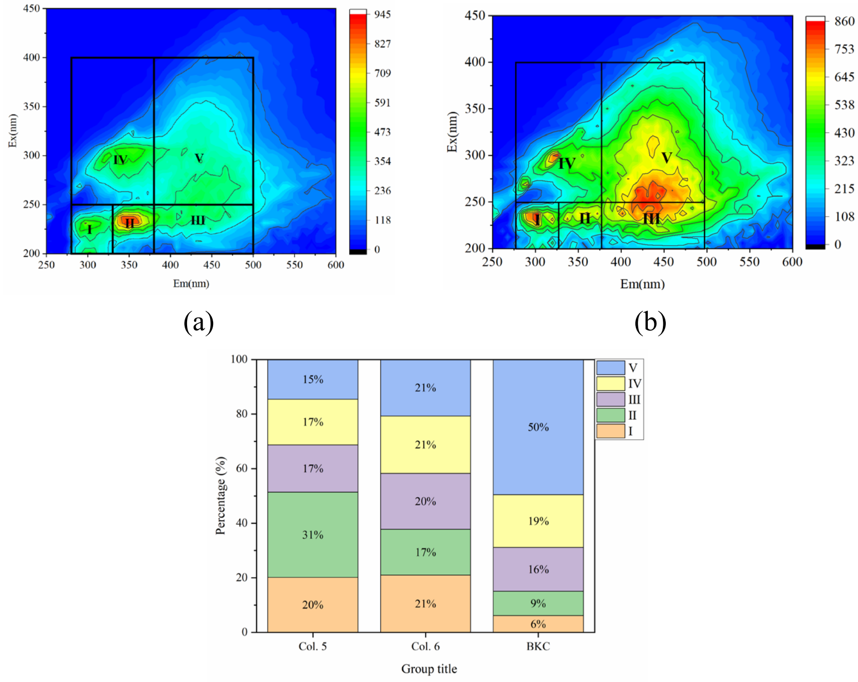

(a) (b)

(c)

Figure S2. The DOM fractions of soil samples. (a) Three-dimensional excitation–emission matrix fluorescence (3D-EEM) of Col. 5. (b) 3D-EEM of Col. 6. (c) Fluorescence regional integration analysis of soil samples. I. Aromatic protein (Ex: 280-330 nm, Em: 200-250 nm), II. tryptophan (Ex: 330-380 nm, Em: 200-250 nm), III. fulvic acid-like substance (Ex: 380-500 nm, Em: 200-250 nm), IV. soluble microbial by-product (Ex: 280-380 nm, Em: 250-400 nm), V. humic acid-like substance (Ex: 380-500 nm, Em: 250-400 nm). Ex: excitation. Em: emission.

1. *HJ 889-2017*; Ministry of Ecology and Environment the People's Republic of China. Soil Quality-Determination of Cation Exchange Capacity(CEC)-Hexamminecobalt Trichloride Solution-Spectrophotometric Method. Available online: https://english.mee.gov.cn/Resources/standards/Soil/Method_Standard4/201801/t20180105_429211.shtml (accessed on 1 February 2018).
2. Wang, Y.; Zhang, X.; Zhang, X.; Meng, Q.; Gao, F.; Zhang, Y. Characterization of spectral responses of dissolved organic matter (DOM) for atrazine binding during the sorption process onto black soil. *Chemosphere* **2017**, *180*, 531–539, https://doi.org/10.1016/j.chemosphere.2017.04.063.
3. *HJ 911-2017*; Ministry of Ecology and Environment the People's Republic of China. Soil and Sediment-Extraction of Organic compounds-Ultrasonic Extraction. Available online: https://codeofchina.com/standard/HJ911-2017.html (accessed on 1 April 2018).
4. *HJ 894-2017*; Ministry of Ecology and Environment the People's Republic of China. Water Quality-Determination of Extractable Petroleum Hydrocarbons (C10–C40) Gas Chromatography. Available online: https://english.mee.gov.cn/Resources/standards/water_environment/method_standard2/201801/t20180105_429205.shtml (accessed on 1 February 2018).
5. Qureshi, A.A.; Kazi, T.G.; Baig, J.A.; Arain, M.B.; Afridi, H.I. Exposure of heavy metals in coal gangue soil, in and outside the mining area using BCR conventional and vortex assisted and single step extraction methods. Impact on orchard grass. *Chemosphere* **2020**, *255*, 126960, https://doi.org/10.1016/j.chemosphere.2020.126960.
6. Zhao, L.; Yan, Y.; Yu, R.; Hu, G.; Cheng, Y.; Huang, H. Source apportionment and health risks of the bioavailable and residual fractions of heavy metals in the park soils in a coastal city of China using a receptor model combined with Pb isotopes. *CATENA* **2020**, *194*, https://doi.org/10.1016/j.catena.2020.104736.
7. *HJ 832–2017;* Ministry of Ecology and Environment the People's Republic of China. Soil and Sediment-Digestion of Total Metal Elements- Microwave Assisted Acid Digestion Method. Available online: https://english.mee.gov.cn/Resources/standards/Soil/Method_Standard4/201708/t20170830_420636.shtml (accessed on 1 September 2017).
8. *HJ 678–2013*; Ministry of Ecology and Environment the People's Republic of China. Water Quality-Digestion of Total Metals-Microwave Assisted Acid Digestion Method. Available online: https://www.codeofchina.com/standard/HJ678-2013.html (accessed on 1 February 2014).
9. Huang, Z.; Chen, Q.; Yao, Y.; Chen, Z.; Zhou, J. Micro-bubbles enhanced removal of diesel oil from the contaminated soil in washing/flushing with surfactant and additives. *J. Environ. Manag.* **2021**, *290*, 112570, https://doi.org/10.1016/j.jenvman.2021.112570.
